# Supplementary material for: CVD-associated SNPs with regulatory potential reveal novel non-coding disease genes
Source: Hum Genomics. 2023 Jul 25;17:69. doi: 10.1186/s40246-023-00513-4 (PMC10369730; doi:10.1186/s40246-023-00513-4)
Supplement: Supplementary file 1 — Additional file 1: Fig. S1. Disease enrichment analysis for protein-coding genes. Bar plots representing per GWAS selected phenotypes enriched for protein coding genes identified with SNEEP. The x-axis shows the -log10 FDR corrected P-value of the disease enrichment analysis performed with the DisGeNET software (usage of DisGeNET similar to method section ‘Identification of disease associated genes using rSNPs', as input the protein-coding genes from the SNEEP result per GWAS are taken) (see also Additional file 2: Table S1). For the GWAS Myocardial ischemia and Aortic stenosis the disease enrichment analysis was not possible, because only 5 and 12 protein coding genes were associated. Fig. S2. Expression of ncRNAs in human MI hearts. Relative RNA expression level of ncRNAs RP11-98F14.11, RPL23AP92, IGBP1P1, and CTD-2383I20.1 in human MI hearts compared to healthy adult hearts (Ctrl). Data are represented as Mean ± SEM; Ctrl: n = 4; MI: n ≥ 9; *P < 0.05 vs. Ctrl. Two-tailed unpaired t-test. MI: Myocardial infarction. Fig. S3. Identification of the best GapmeRs in hiPSC-CMs. A Relative RNA expression level of RP11-98F14.11 in hiPSC-CMs treated with GM RP11-98F14.11 #1, #2, #3, #4 or GM GapmeR Ctrl. Data are normalized to hiPSC-CMs expressing GM Ctrl. Data are represented as Mean ± SEM; n = 3; *P < 0.05 vs. GM Ctrl. B Relative RNA expression level of RPL23AP92 in hiPSC-CMs treated with GM RPL23AP92 #1, #2, #3, #4 or GM Ctrl. Data are represented as Mean ± SEM; n = 3; *P < 0.05, **P < 0.01 vs. GM Ctrl. C Relative RNA expression level of IGBP1P1 in hiPSC-CMs treated with GM IGBP1P1 #1, #2, #3, #4 or GM Ctrl. Data are represented as Mean ± SEM; n = 3; *P < 0.05, **P < 0.01 vs. GM Ctrl. D Relative RNA expression level of CTD-2383I20.1 in hiPSC-CMs treated with GM CTD-2383I20.1 #1, #2, #3, #4 or GM Ctrl. Data are represented as Mean ± SEM; n = 3; *P < 0.05 vs. GM Ctrl. Two-tailed unpaired t-test. Fig. S4. Characterization of CTD-2383I20.1 in hiPSC-CMs. Relative RNA express [file 40246_2023_513_MOESM1_ESM.pptx]

## Slide 1
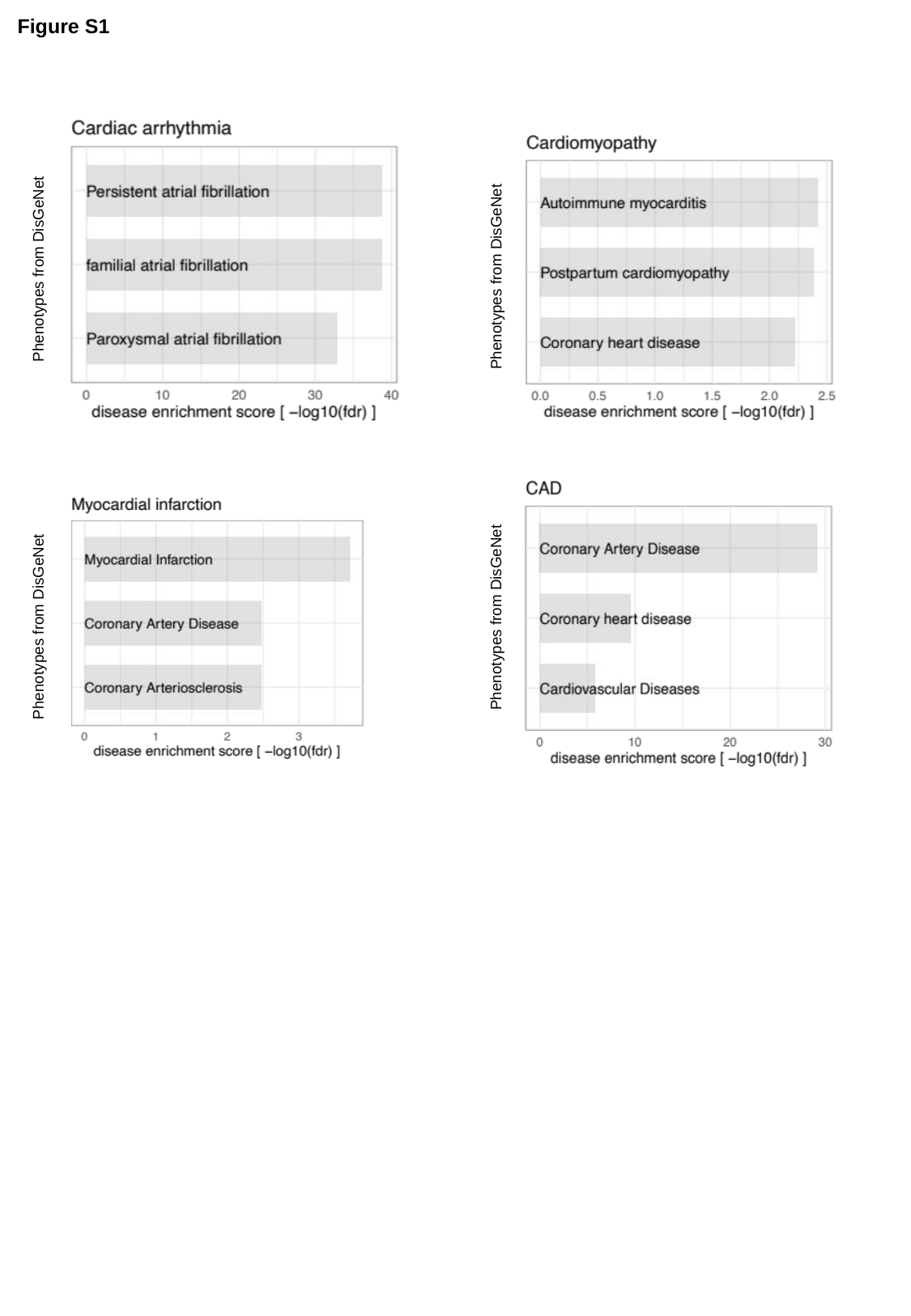

Figure S1
Phenotypes from DisGeNet
Phenotypes from DisGeNet
Phenotypes from DisGeNet
Phenotypes from DisGeNet

## Slide 2
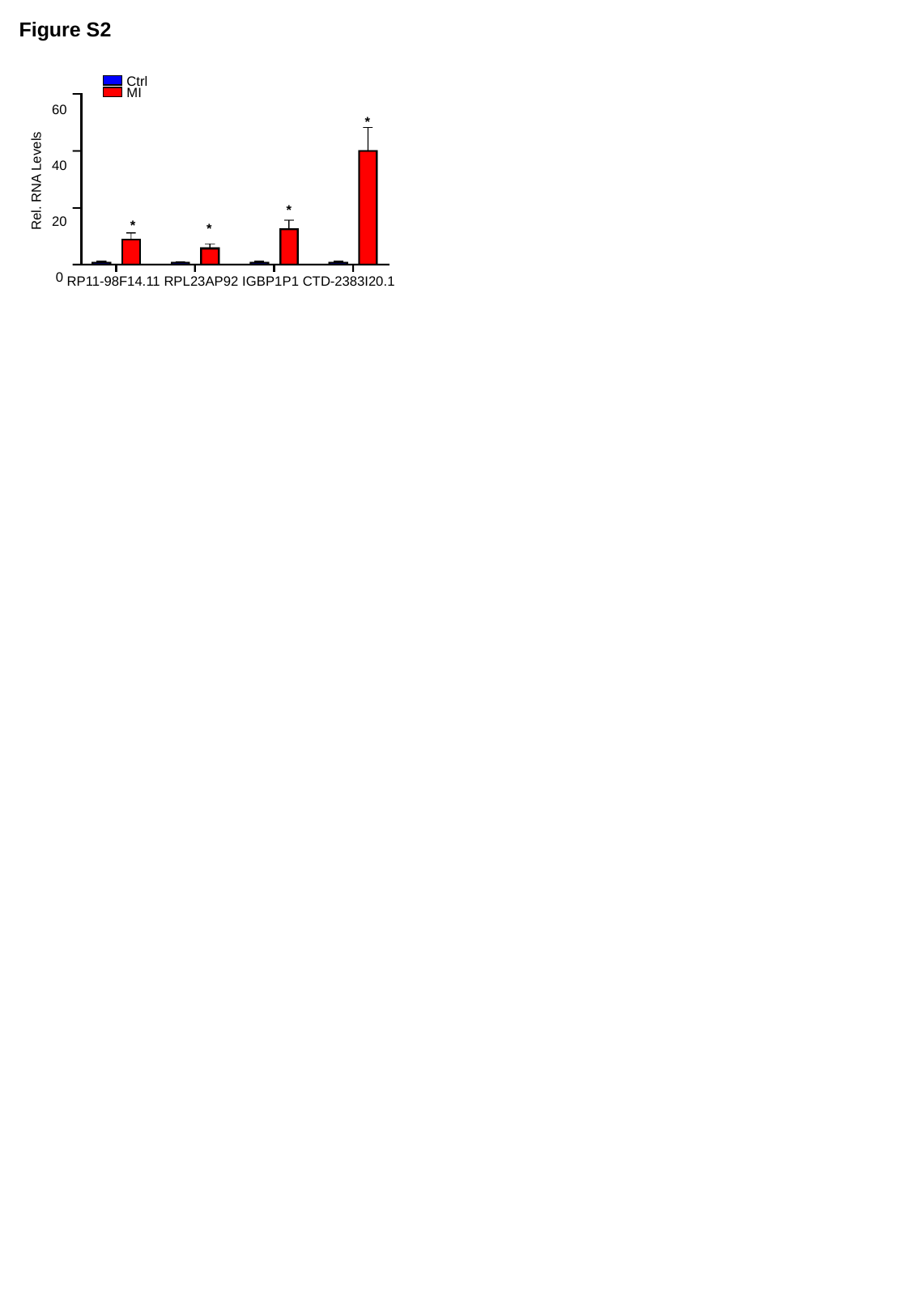

Figure S2
60
40
20
0
Rel. RNA Levels
*
*
*
RP11-98F14.11 RPL23AP92 IGBP1P1 CTD-2383I20.1
Ctrl
MI
*

## Slide 3
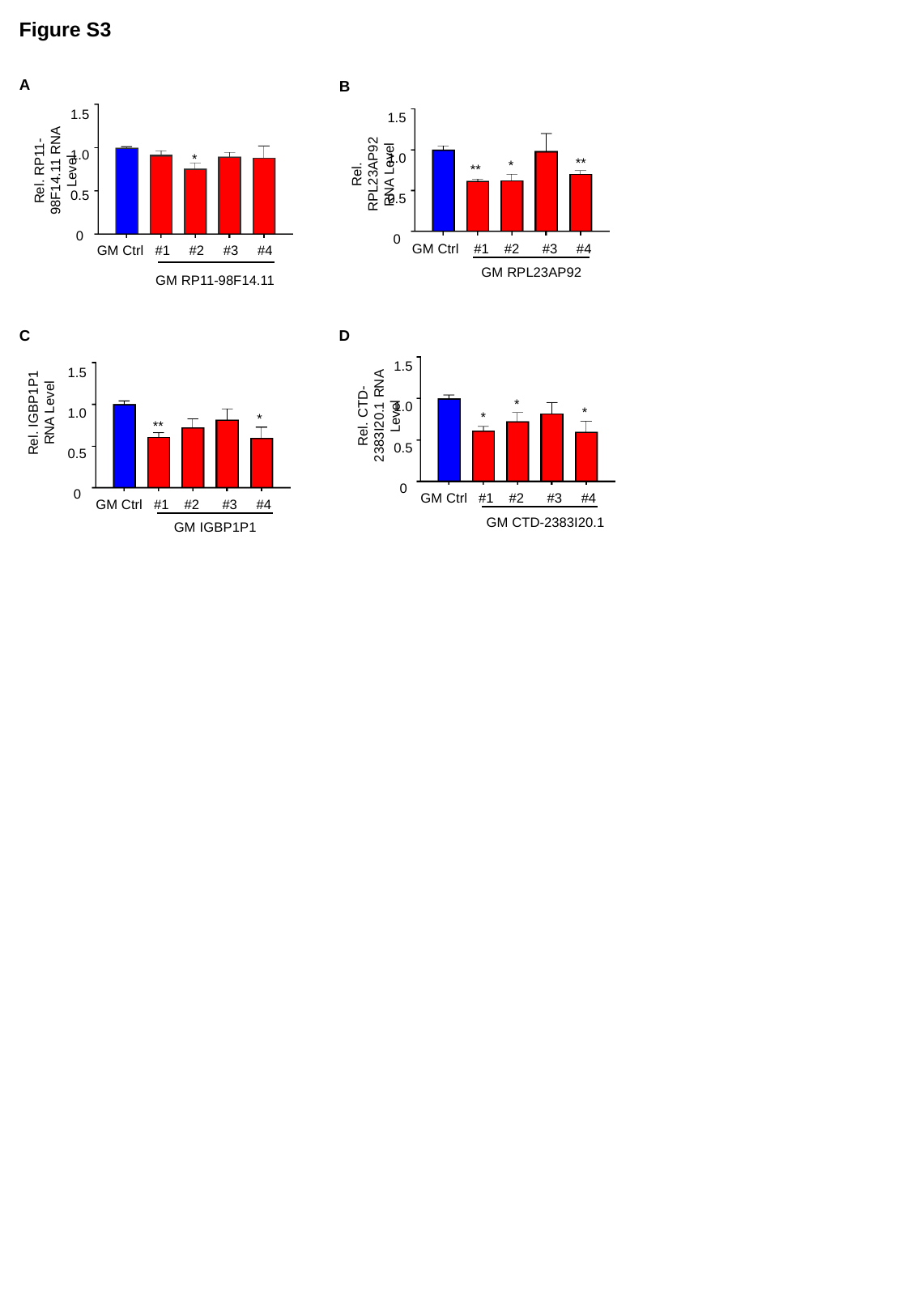

Figure S3
A
B
1.5
1.0
0.5
0
Rel. RP11-98F14.11 RNA Level
*
GM Ctrl #1 #2 #3 #4
GM RP11-98F14.11
1.5
1.0
0.5
0
Rel. RPL23AP92 RNA Level
**
*
**
GM Ctrl #1 #2 #3 #4
GM RPL23AP92
C
D
1.5
1.0
0.5
0
Rel. CTD-2383I20.1 RNA Level
*
*
*
GM Ctrl #1 #2 #3 #4
GM CTD-2383I20.1
1.5
1.0
0.5
0
Rel. IGBP1P1 RNA Level
*
**
GM Ctrl #1 #2 #3 #4
GM IGBP1P1

## Slide 4
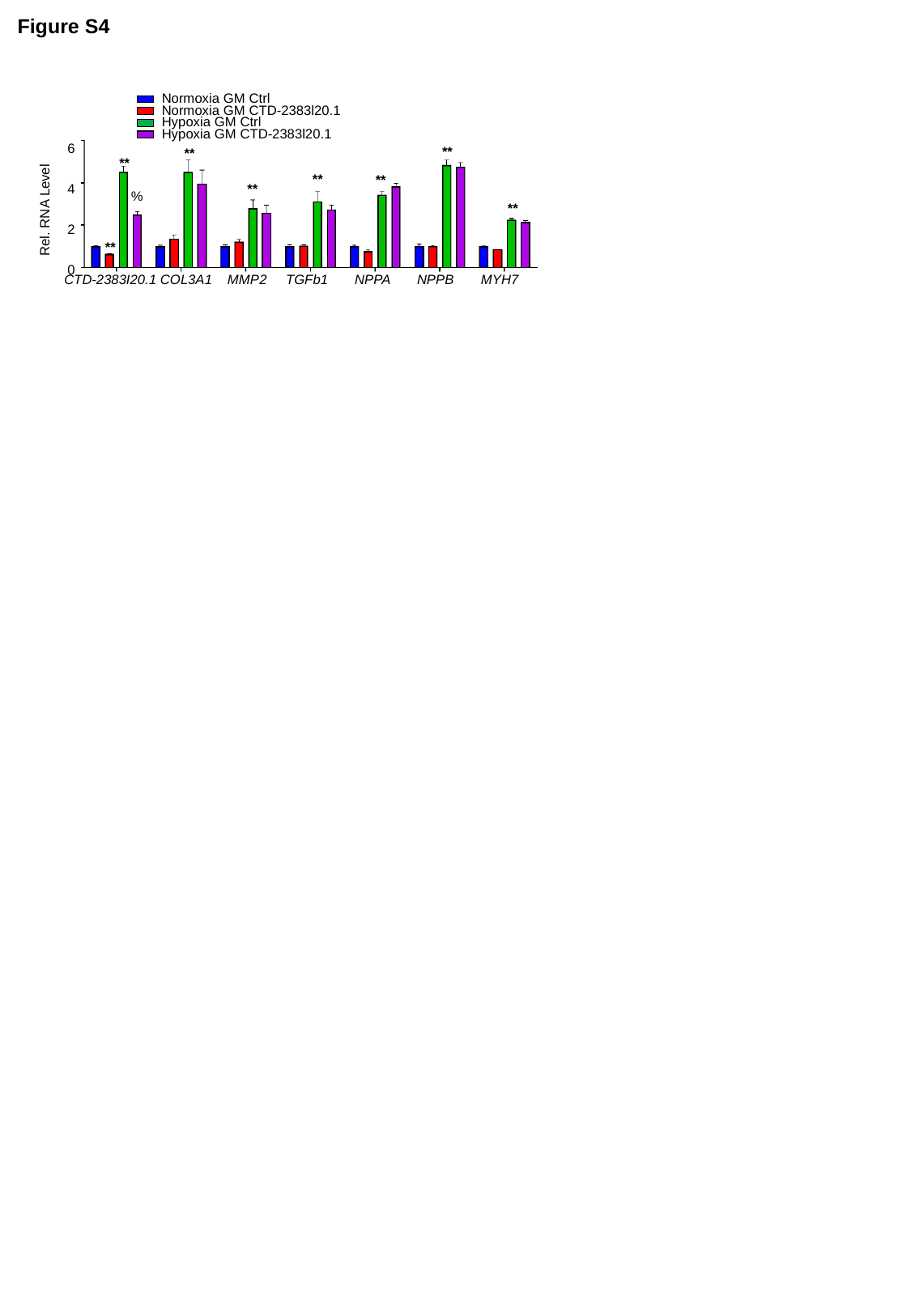

Figure S4
Normoxia GM Ctrl
Normoxia GM CTD-2383l20.1
Hypoxia GM Ctrl
Hypoxia GM CTD-2383l20.1
6
4
2
0
Rel. RNA Level
**
**
**
**
**
**
%
**
**
CTD-2383I20.1 COL3A1 MMP2 TGFb1 NPPA NPPB MYH7

## Slide 5
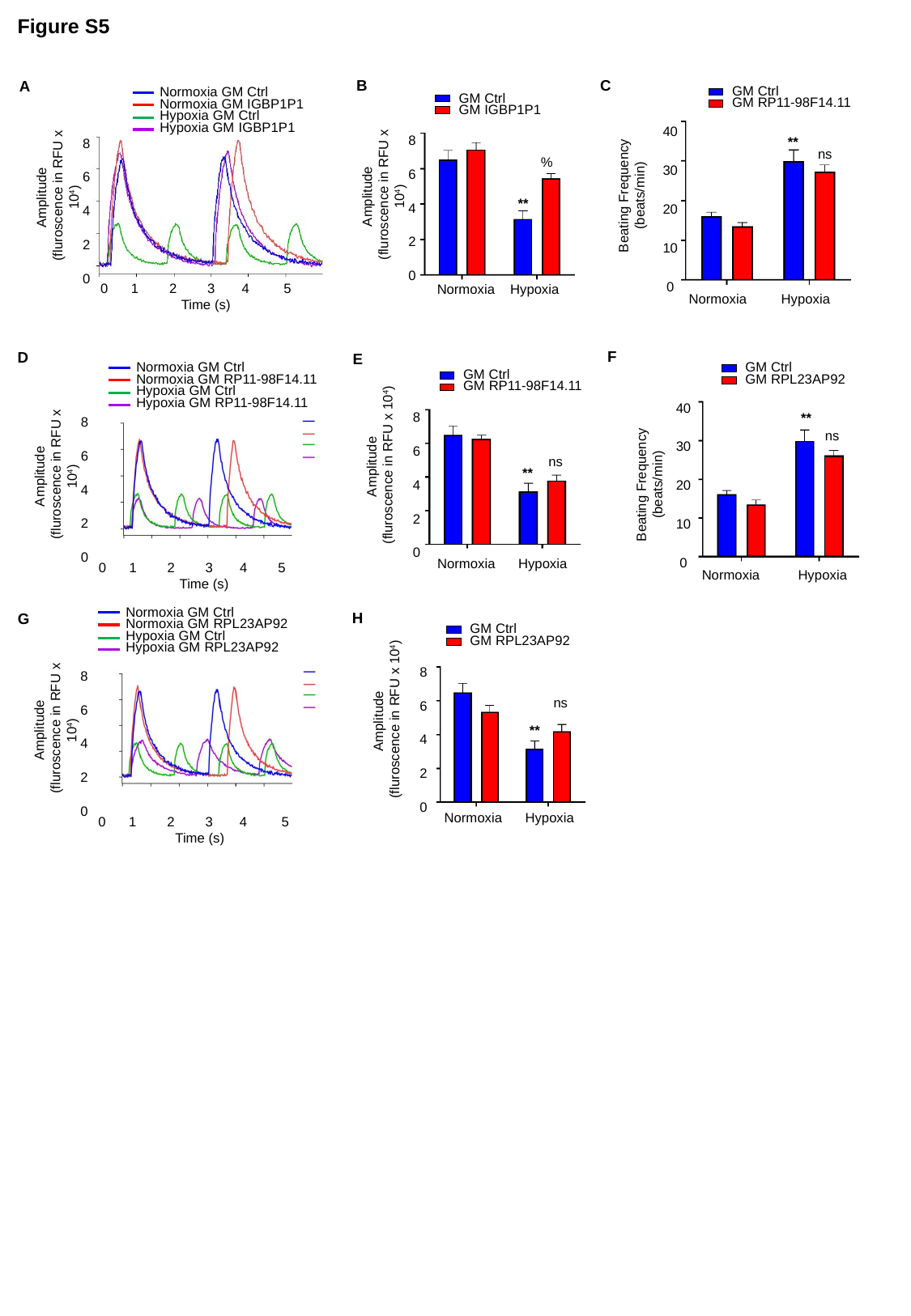

Figure S5
B
C
A
GM Ctrl
GM RP11-98F14.11
40
30
20
10
0
Beating Frequency (beats/min)
Normoxia Hypoxia
**
ns
Normoxia GM Ctrl
Normoxia GM IGBP1P1
Hypoxia GM Ctrl
Hypoxia GM IGBP1P1
0 1 2 3 4 5
 Time (s)
Amplitude
 (fluroscence in RFU x 104)
8
6
4
2
0
GM Ctrl
GM IGBP1P1
Amplitude
 (fluroscence in RFU x 104)
8
6
4
2
0
Normoxia Hypoxia
%
**
F
D
E
GM Ctrl
GM RPL23AP92
40
30
20
10
0
Beating Frequency (beats/min)
Normoxia Hypoxia
**
ns
Normoxia GM Ctrl
Normoxia GM RP11-98F14.11 Hypoxia GM Ctrl
Hypoxia GM RP11-98F14.11
GM Ctrl
GM RP11-98F14.11
Amplitude
 (fluroscence in RFU x 104)
8
6
4
2
0
Normoxia Hypoxia
**
ns
Amplitude
 (fluroscence in RFU x 104)
8
6
4
2
0
0 1 2 3 4 5
 Time (s)
Normoxia GM Ctrl
Normoxia GM RPL23AP92
Hypoxia GM Ctrl
Hypoxia GM RPL23AP92
H
G
GM Ctrl
GM RPL23AP92
Amplitude
 (fluroscence in RFU x 104)
8
6
4
2
0
Normoxia Hypoxia
**
ns
Amplitude
 (fluroscence in RFU x 104)
8
6
4
2
0
0 1 2 3 4 5
 Time (s)
